# Exploratory Assessment of Iron- and Sulfate-Reducing Bacteria in Sediments Co-Contaminated with Metals and Polycyclic Aromatic Hydrocarbons

**DOI:** 10.3390/microorganisms14040885

**Published:** 2026-04-15

**Authors:** Gloria Patricia Johnston, Daniel J. Lisko, Carl G. Johnston

**Affiliations:** Department of Chemical and Biological Sciences, Youngstown State University, Youngstown, OH 44555, USA; gpjohnston@ysu.edu (G.P.J.); djlisko01@gmail.com (D.J.L.)

**Keywords:** sulfate reduction, *Geobacter*, cryptic sulfur, iron reduction

## Abstract

Rivers contaminated with metals and petroleum hydrocarbons, such as polycyclic aromatic hydrocarbons (PAHs), are still a problem that threatens aquatic ecosystem function. This study describes iron- and sulfate-reducing bacteria, principal drivers of anaerobic organic matter decomposition in aquatic sediments. A polyphasic approach, including culture-dependent, i.e., enumeration by Most Probable Number (MPN), and independent, Sanger and Next Generation Sequencing (NGS) techniques, as well as analytical geochemical analyses, was employed. This study found exceptionally high levels of metals (Al, Mn, Zn, and Pb), PAHs, and sulfates compared to typical freshwater environments, likely due to co-contamination from past petroleum and steel production waste. Microbial communities were dominated by the Thermoproteobacteria. Analysis of the iron-reducing community determined that *Geobacter*, critical for degrading organic matter using iron, manganese, or arsenic, was the most prevalent genus. Additionally, the presence of diverse groups involved in sulfur cycling, represented by *dsrAB* genes, high numbers of viable sulfate reducers, a higher abundance of *Geobacter*, and high levels of sulfate and iron suggests that the cryptic sulfur cycle (CSC) may be operational in this system. In addition, sulfate and iron reducers are known to enhance biodegradation of organic pollutants in the presence of metal oxides and sulfate, and thus warrant further investigation in this co-contaminated system.

## 1. Introduction

Sediment contamination with polycyclic aromatic hydrocarbons (PAHs) and metals is a common legacy of industrial activities worldwide. PAHs are a group of recalcitrant organic contaminants present in anoxic environments because degradation is often limited by available alternative electron acceptors, such as sulfate, nitrate, Fe (III), and Mn (IV) (Zhao et al., 2026 [1]). Metals are also extensive, persistent pollutants affecting numerous terrestrial and aquatic environments (Hu et al. 2024 [2]), with lead (Pb), chromium (Cr), cadmium (Cd), zinc (Zn), and arsenic (As) being the most ubiquitous (Ondrasek et al. 2025 [3]).

The coexistence of multiple pollutants is a discriminant anthropogenic marker and affects soil ecology, ecosystem function, nutrient cycling, and potential for biodegradation. Extensive research has shown different outcomes from different systems regarding whether and how microbial communities respond to PAHs (Johnston et al. 2015 [4]; Castro et al. 2022 [5]). Under the pressure of metal pollution, microbial communities can become resistant and/or shift functional properties (e.g., from ammonia oxidizers to nitrifiers). For instance, soils with mixed contaminants, including per- and polyfluoroalkyl substances (PFASs), heavy metals, and PAHs, despite reducing the richness and diversity of microbial communities, appear to also demonstrate adaptation or resistance, whereby some genera tolerate the combined stress exerted upon them (Zhang et al. 2024a [6]; Gou et al. 2026 [7]).

In sediments and soils, Fe (II) and sulfide, products of microbial iron and sulfate reduction, respectively, are recognized as key environmental factors that shape local communities (Wunder et al. 2021 [8]). Sulfate-reducing bacteria (SRB) are a metabolically flexible group responsible for mineralization of organic matter in anaerobic environments, accounting for 50% of organic carbon mineralization in marine sediments (Diao et al. 2023 [9]), facilitated by high sulfate availability (~20 mM). SRB play a key role in heavy metal immobilization (Jha et al. 2024 [10]), metal removal from wastewaters (Zampieri et al. 2020 [11]), and can enhance bioremediation by forming synergistic associations with other microorganisms (Kushkevych et al. 2021 [12]). Iron-reducing bacteria (FeRB) are another metabolically diverse group of microorganisms that determine the rate of carbon cycling in anaerobic environments (Lovley et al. 2004 [13]), contributing to 45% to 85% of organic sequestration in sediments and soils (Fan et al. 2018 [14]). FeRB are involved in metal precipitation (Di et al. 2025 [15]), mineralization of PAHs (Castro et al. 2022 [5]), and dehalogenation of chlorophenols (Zu et al. 2025 [16]).

In anoxic sediments, sulfate, Fe (III), and Mn (IV) are important terminal electron acceptors coupled to oxidation of organic matter and oxidation of hydrogen sulfide by reactive iron (III) (hydr)oxides, the most bioavailable form of iron (Jørgensen, 2021 [17]). If the flux of iron is higher than the flux of reduced sulfur, (1) sulfate reduction might appear to be outcompeted by iron-reducing microorganisms (Lovley and Phillips, 1987 [18]), and (2) hydrogen sulfide (formed by microbial sulfate reduction) can be immediately reoxidized by Fe (III). This combined process, called “cryptic″ sulfur cycling (i.e., reoxidation or precipitation of produced sulfide at a small or undetectable pool size), as reviewed in Grijalva-Rodriguez et al. 2025 [19] and referenced therein, has been studied mostly in marine sediments. For instance, Wunder et al. 2021 [8] observed in Antarctic sediments that sulfate reduction was active at the same time as iron reduction, indicating that sulfate reduction was “masked″ by reoxidation of sulfide, which, in turn, allows increased sulfate reduction. In addition, Jørgensen et al. (2019 [20]) indicated that in marine sediments, where sulfate is normally present in high concentrations in the water column and diffuses to sediments, iron reduction is constrained by the reactivity and lower availability of ferric iron compared to sulfate. Most recently, the sulfur and iron cycles, and the interplay of both groups of prokaryotes associated with these cycles, have been elucidated. Chen et al. (2025 [21]) characterized the metabolism of coupling sulfide oxidation with extracellular Fe (III) reduction (a strict anoxic process) in 37 phyla, indicating that this cryptic process is widely distributed in marine sediments, wetlands, and aquifers. Nevertheless, there is a scarcity of studies that have investigated both iron- and sulfate-reducing bacteria in freshwater lake sediments (Ruiz-Blas et al. 2025 [22], Zhao et al. 2021a [23]; Heinrich et al. 2022 [24]), and even fewer in riverine sediments (Wang and Pan, 2025 [25]; Bai et al. 2023 [26]).

In urban settings, where riparian vegetation is often the only remnant native vegetation, riverbanks are targets of ecological restoration programs, including bioremediation. Previous reports on this system showed that bacterial communities were influenced by hydrocarbon contamination (Johnston and Leff 2015 [27]) and appeared to have potential for degradation of PAHs (Johnston et al. 2016 [28]). In addition, PLFA analysis showed that approximately 1/3 of the microbial community consisted of sulfate-reducing bacteria (Pratt et al. 2012 [29]), and earlier metal analyses (Farnham et al. 2012 [30]) indicated high-level concentrations of iron. The goal of this exploratory investigation was to characterize the distribution of metals, sulfate, and PAHs, as well as native SRB and FeRB communities to the genus/species level, to predict the potential metabolism of organic contaminants with metal and sulfur oxides as terminal electron acceptors in riverbank sediments, using culture and culture-independent techniques. The results of this study will allow for a better assessment of the bioremediation of these and similarly contaminated sites.

## 2. Methods

### 2.1. Study Site, Sampling, and Physicochemical Analyses

**Figure 1:**
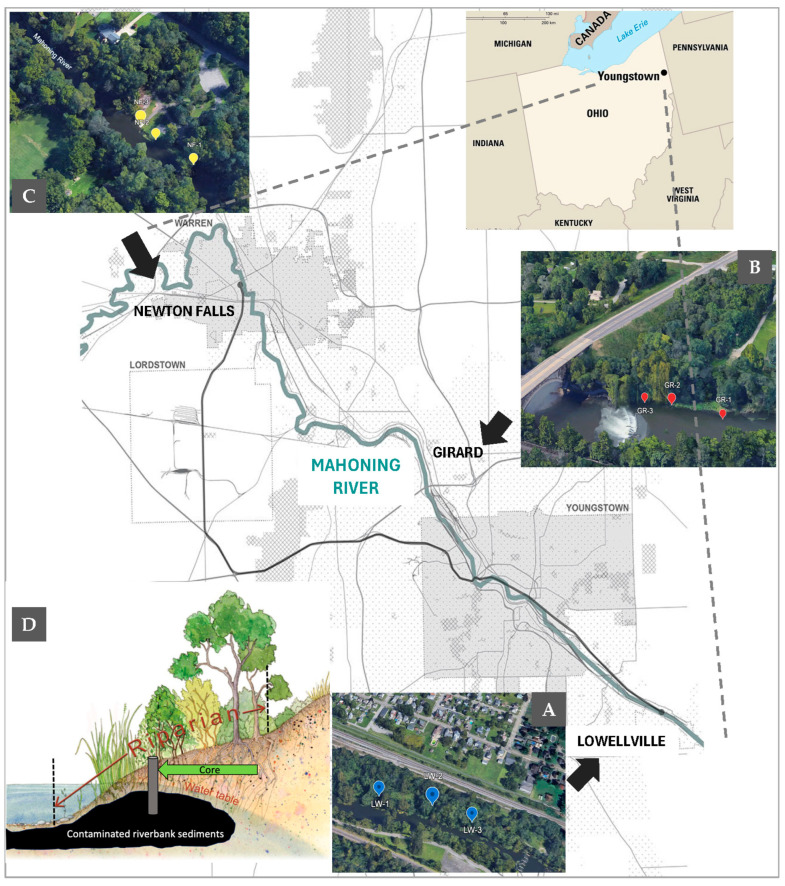
Sampling sites along the Mahoning River, Lowellville (**A**), high contamination, Girard (**B**), high contamination, and Newton Falls (**C**), low contamination, where sediment cores were taken ~100 m apart. (**D**) A representational cross-section of core sampling in the riparian zone where the contaminated subsurface riverbank sediments were situated.

The Mahoning River, a tributary of the Ohio River watershed, supported one of the biggest steel industries worldwide from the 1700s until the mid-1970s, leaving high concentrations of metals and hydrocarbons, among other pollutants, in the sediment. The booming industries used the river as a source of cooling and disposal along the riparian zone (riverbanks). The iron and steel industry generated large amounts of toxic compounds, including PAHs, cyanides, phenols, BTEX, and dissolved metals. Accordingly, mixed waste (~180,000 kg/day) of the pollutants and metals were released into the river and were simultaneously deposited from upstream in Leavittsburgh/Newton Falls (Figure 1), where fewer industries were located, to downstream in Lowellville, where most of the steel industry settled. Compared to bigger river systems (i.e., the Hudson River), contamination in this site (51 km) was the result of a more intensive use of the river water, resulting in a stretch of heavy contamination in locations downstream. Nine sediment cores were taken from the riparian zone of the Mahoning River from two heavily polluted sites (LW and GR) and one less polluted site (NF), which is upstream and did not receive such high input of mixed wastes. At each location (LW, GR, and NF), riverbanks showed similar vegetation but were heterogeneous with respect to their hydrodynamics (water height, discharge, and flow). Sediment cores were collected 1.5 to 3 m below ground surface, depending on the accessibility of the riverbanks. As in many riparian zones, roots, fallen trees, and other vegetation covered the surroundings, limiting exactly where sediments could be sampled. Once a clear site was identified, the cores were retrieved using a manual auger device (AMS, American Falls, ID, USA) with sterile stainless-steel liners (diameter = 5 cm, length = 15 cm), replaced after each collection. The cores were collected approximately 100 m apart from each other (Figure 1) and were immediately capped (using stainless steel plates and plastic caps), sealed in plastic bags, and transported on ice to the laboratory. In the laboratory, the bagged cores were sealed under anaerobic conditions and kept at −80 °C. For analysis, the cores were thawed, both ends were discarded, and the inner core was homogenized under nitrogen inside an anaerobic bag (Sigma, St. Louis, MO, USA) using sterile techniques. Three different sediment subsamples from each core were partitioned for geochemical data, culturing, and DNA extraction.

### 2.2. Sediment Geochemistry

PAHs were extracted by a modification of a sonication-assisted extraction method set forth in USEPA (2015a [31]), Method 3550C, and quantified by Gas Chromatography Mass Spectrometry, as described in Johnston et al. 2015 [4]. Total nitrogen (TN), total carbon (TC), sulfate (TS), pH, sediment moisture content (MC), and organic matter (OM) were determined by standard methods and described elsewhere (Johnston et al. 2015 [4]). Metal concentrations were determined by microwave digestion (USEPA, 2015b [32]), using trace metal grade nitric acid (Fisher Scientific, Pittsburgh, PA, USA). All other reagents were also of analytical grade. Ultrapure water (Milli-Q System, Millipore, Billerica, MA, USA) was used for the preparation of solutions and dilutions. The glassware was cleaned by soaking in 10% HCl (Fisher Scientific, Pittsburgh, PA, USA) for at least 24 h and rinsed liberally with ultrapure water. Approximately 0.1 g of homogenized dried sediment was extracted using 10 mL of trace metal nitric acid. After microwave digestion, the digested products were adjusted to 50 mL using ultrapure water. The samples were diluted and analyzed for Be, Al, Cr, V, Mn, Fe, Co, Ni, Cu, Zn, Ga, As, Se, Sr, Cd, Ag, Ba, Pb, Tl, and U by inductively coupled plasma mass spectrometry (iCAP ICP-MS, ThermoScientific, Waltham, MA, USA). Quality controls were analyzed in accordance with method requirements. Recoveries for standard reference materials (NIST Buffalo River No 2704, Sigma-Aldrich, St. Louis, MO, USA) were between 85 ± 5% for most metal concentrations. The element standard solution (SPEX CertiPrep, Metuchen, NJ, USA) used for calibration was prepared by diluting a stock solution of 10 mg L^−1^.

### 2.3. Bacterial Enumeration

The total number of bacteria was determined in a sediment subsample preserved with equal parts of paraformaldehyde (8% final concentration) and phosphate-buffered saline. Briefly, after sonication, samples were filtered (0.22 μm) and stained with 4,6- diamidino-2-phenylindole (Sigma-Aldrich, St. Louis, MO, USA), 1 mg /mL final concentration, and enumerated under an epifluorescence microscope (Olympus DP71, Evident Corporation, State College, PA, USA) as previously described (Johnston and Leff, 2015 [27]). SRB were determined by most probable numbers (MPNs) from slurry solutions in a 96-well microtiter plate (Johnsen et al. 2002 [33]). All following reagents were laboratory-grade purchased through Sigma-Aldrich (St. Louis, MO, USA). Modified reducing medium (Fortin et al. 1996 [34]) consisting of (in g/L): Bacto™-Tryptone, 10, 5.9 mL 60% Na-lactate, MgSO_4_·7H_2_O, 2.0, FeSO_4_·7H_2_O, and 0.5, Na_2_SO_3_, along with a reducing agent (RAS) made with 7.5 g L^−1^ ascorbic acid and 7.5 mL L^−1^ thioglycollic acid), adjusted to pH 7.5 with 2 M NaOH, was used as the growth medium. Ten-fold serial dilutions were performed before inoculation in wells containing 200 μL media. Sterile controls (using ultrapure autoclaved water) were also included. The MPN plates were incubated anaerobically using a GazPak EZ Pouch System (BD Technologies, Franklin Lakes, NJ, USA) at 37 °C until growth was observed. Positive wells (by presence of black precipitates) were scored after 12 h and 2 days of incubation time, using statistical tables.

### 2.4. Bacterial, Archaeal and Iron-Reducing Community Composition by NGS

Total DNA was extracted from 0.25 g of previously thawed sediment using the Power Soil DNA Isolation Kit (MoBio, Laboratories, Carlsbad, CA, USA), according to the manufacturer’s instructions with minor modifications. Duplicate DNA extracts from each subsample were pooled and stored at −20 °C until analysis. The quality and size of the DNA were checked by electrophoresis on 1% agarose gels.

Bacteria, archaeal, and FeRB were analyzed by amplicon sequencing data (Illumina MiSeq (Illumina Inc., San Diego, CA, USA) pair-end 300 bp. All primers were purchased from Integrated DNA Technologies, Inc. (Coralville, IA, USA). The primer pair 8F (5′-AGAGTTTGATCATGGCTCAG-3′) and 1492R (5′-GGCTACCTTGCCACGACTTC-3′) (Zhang et al. 2008 [35]) and the primers 519F (5′-CAGCCGCCGCGGTAA) and 915R (5′-GTGCTCCCCCGCCAATTCCT) (Yan et al. 2018 [36]) were chosen to target bacteria and archaea, respectively. The primers Geo564F (5′-AAGCGTTGTTCGGAWTTAT-3′) and Geo840R (5′-GGCACTGCAGGGGTCAATA-3′) (Holmes et al., 2002 [37]; Cummings et al. 2003 [38]) were used to detect Geobacteraceae and other FeRB families. The size of the amplicons was checked by electrophoresis on 1% agarose gels. Amplicons for different samples were pooled in equimolar ratios for sequencing analyses, and library preparation was established by Case Western Reserve University (Cleveland, OH, USA). After final reads were provided, raw sequences were processed using the QIIME 1 pipeline (Caporaso et al. 2010 [39]). Paired-end sequences were joined and removed from analysis if they were <200 nucleotides, had a quality score < 25, contained <6 ambiguous characters, or had a homopolymer length < 6. Usearch software (Edgar 2010 [40], version v5.2.236) was used for chimera screening and filtering using de novo and reference-based methods, using the Silva database (Silva119_release_aligned_rep_files.tar.gz) (Quast et al. 2012 [41]). Quality-filtered sequences were clustered into operational taxonomic units (OTUs) with 97% similarity. A representative sequence was then selected for each OTU by selecting the most abundant sequence among the OTUs. Representative sequences were aligned to the Silva database (Quast et al. 2012 [41]) using MUSCLE (Edgar 2004 [42]), and taxonomy was assigned using BLAST (2.2.22 version). Rarefaction was performed on all samples to standardize sequencing depth, using a minimum subsampling depth (determined by the sample with the lowest number of sequences) at 100 replicates.

### 2.5. Clone Library Construction and Sequencing Analysis of Dissimilatory Sulfite Reductase (dsrAB)

Earlier investigations in Mahoning riverbank sediments using phospholipid fatty acid analyses (Pratt et al. 2012 [29]), Sanger sequencing (Johnston and Leff 2015 [27]), and enumeration of viable bacteria reported the presence of SRB. The *dsrAB* gene, which encodes the enzyme dissimilatory sulfite reductase, was chosen because it is a key enzyme catalyzing the reduction of sulfite to sulfide during anaerobic respiration whenever sulfate is used as the terminal electron acceptor (conversely, it works in reverse in microbial sulfur oxidation) (Ferreira et al. 2022 [43]). Thus, the sulfate-reducing community was assessed by investigating the distribution of the *dsrAB* functional gene to characterize the sulfur-associated microbiome community. From each sediment core (total of 9 cores), DNA was extracted, and the primer pair DSR1F (5′-AC[C/G]CACTGGAAGCACG-3′) and DSR4R (5′-GTGTAGCAGTTACCGCA-3′) was used to amplify the *dsrAB* gene (Jiang et al. 2009 [44]). Negative controls were performed using nuclease-free water. Positive controls were performed using SRB from an anaerobic culture, using specific SRB-reducing media (Fortin et al. 1996 [34]). PCR consisted of an initial denaturation at 94 °C for 1 min, followed by 30 cycles of 94 °C for 1 min, 55 °C for 1 min, and 72 °C for 90 s, with a final extension at 72 °C for 10 min. Amplified PCR products were separated by 1% (*w*/*v*) agarose gel electrophoresis to verify the size of the fragments amplified. The PCR products were cloned into competent *Escherichia coli* cells using the StrataClone PCR Cloning Kit TA cloning kit (Agilent Technologies, Santa Clara, CA, USA), following the manufacturer’s instructions with minor modifications. Approximately 400 clones were processed, and a subset of 110 clones (from all sites combined) were chosen to be sequenced at the University of Kentucky Advanced Genetic Technologies Center, Lexington, KY, USA. The sequences were submitted to the Ribosomal Database Project II (https://bio.tools/rdp, accessed on 3 January 2023) to detect the presence of chimeric artifacts. After quality filtering, only 103 sequences were curated by using two parallel BLASTX searches (https://blast.ncbi.nlm.nih.gov/Blast.cgi?LINK_LOC=blasthome&PAGE_TYPE=BlastSearch&PROGRAM=blastx, accessed on 14 February 2023). For the construction of the phylogenetic tree, reference sequences (Zhu et al. (2022 [45])) were included. All unknown sequences were translated into protein and aligned using MUSCLE v5. No gaps were manually removed (IQ-TREE 2 handles gap-rich sites internally). The phylogenetic tree was constructed using IQ-TREE 2 (Minh et al. 2020 [46]), with a LG substitution model (Kalyaanamoorthy et al. 2017 [47]).

### 2.6. Statistical Analysis

Unrotated principal component analysis (PCA) of untransformed geochemical data (pH, TC, TN, TS, MC, OM, PAHs, and metals) was performed to establish links among environmental variables. A one-way multivariate analysis of variance (MANOVA) was used to determine if there were significant differences among sampling sites in pH, TC, TN, TS, MC, OM, PAHs, metals, T-RFs, and bacteria counts. Pearson correlation coefficients for the relationships between those variables and Shannon–Wiener diversity indexes of FeRB and *dsrAB* sequences from sediment samples at each site were also calculated. Good’s coverage estimator was used to measure completeness of the *dsrAB* clone library by the following formula: C = 1 − *n*_1_/*N*, where n is the number of phylotypes that occurred only once (singletons), and N is the total number of clones recovered. Alpha diversity (OTU-based) for FeRB was calculated on rarefied sequences using Chao1 (richness), the Shannon–Wiener index, and Good’s coverage. Beta diversity for FeRB was computed using weighted UniFrac distances to compare microbial communities based on both the fraction of unique branch lengths observed in pairs for the phylogenetic tree and the relative abundance for each microbial community. Jackknifing was incorporated by resampling 10 times, with a sequencing depth of 100 sequences per sample. NMDS was performed to compare beta diversity among the sediment cores. Statistical analyses were performed using IBM SPSS Statistics 20 for Windows (IBM Corp., Armonk, NY, USA). Raw sequence data are available in the Sequence Read Archive at the National Center for Biotechnology Information under study PRJNA389485, with accession numbers SAMN200607–200612.

## 3. Results

### 3.1. Soil Geochemistry

The average pH of the sediment cores from LW and GR were similar (ranging from 6.8 to 7.3), while the sediment cores from NF exhibited values below neutral (Table 1). The moisture content was homogeneous in the sediment cores from each location, yet sediments collected upstream (NF) had much lower moisture content (~25%). Visual observations (while sampling and performing analyses) revealed that sediments from LW and GR were oily, black, and viscous, with fewer clay particles, while sediments collected from NF had more clay content, with streaks of iron deposition (rusty color), indicators of the complex mixture of total petroleum hydrocarbons (TPHs). Measurements of total organic carbon and organic matter content were higher for the LW and GR sediments (~9%) compared to the NF sediment (1.5%), corroborating this observation.

PAHs, the most recalcitrant subset of the TPHs, are the most resistant to biodegradation due to the inherent stability of aromatic rings (Zeneli et al. 2019 [48]). Thus, this study focused on measuring PAHs, since these compounds represent the greatest long-term environmental risk due to their persistence in low-oxygen environments. PAH concentrations showed variable distribution among the cores from each site, yet they were much higher in LW than GR and NF sediments (Table 1). PAHs tend to strongly adhere to clays and organic matter on the surface, where clay can absorb them (Saeedi et al. 2012 [49]), yet in this study, PAHs were generally much higher at deeper depths (high correlation), indicating that earlier deposition of contamination was much higher compared to levels that accumulated more recently after the closure of the local steel industry.

Total sulfate was quite variable in each core from LW (ranging from 1200 to 12,890 μM), yet was 15 times higher than GR (ranging from 300 to 830 μM) and NF sediments (ranging from 830 to 1800 μM). Total nitrogen was uniform for all the cores and sites (0.2%), while the average total carbon from the sediment cores sampled from LW and GR (~8.5%) was statistically significantly different from NF (1%).

Most metal concentrations in sediments downstream (LW and GR) were 5 to 10 times higher (Table 2) than metal concentrations in sediments collected upstream with heavy contamination (NF). All metals were highly significantly correlated (*p* < 0.01) with total metal concentrations, except for Be, Al, Ga, Se, and U, which did not correlate with total metal concentrations. Significant Pearson correlations were found between Fe and Cr (r = 0.75, *p* < 0.05), Fe and Mn (r = 0.95, *p* < 0.01), Fe and Co (r = 0.89, *p* < 0.01), Fe and Zn (r = 0.79, *p* < 0.05), Al and Mn (r = 0.89, *p* < 0.01), Al and Pb (r = 0.91, *p* < 0.01), and Mn and Pb (r = 0.82, *p* < 0.01).

Among the metals, Fe concentrations were 20 times higher in the sediment cores from LW and GR (300,000 μg g^−1^) than in the cores from NF (14,000 μg g^−1^), and much higher than what has been reported for similar heavily contaminated sites. Fe, Al, Mn, and Zn were also more abundant than other metals at each site (Table 2). In addition, the sediments collected from GR had the highest average concentrations for arsenic (60 μg g^−1^) in comparison to sediments from LW (23 μg g^−1^) and NF (8 μg g^−1^), exceeding regulatory guidelines by 50%. Higher arsenic concentrations can be found in iron and manganese ores and/or as a by-product of smelting (USEPA, 2026a [50]). Thus, metal contamination was evaluated by comparing sediment quality guidelines (USEPA, 2026b [51]). Accordingly, the sediment cores sampled from GR were heavily polluted with Cd, Cr, Cu, Pb, and Zn, while the sediment cores from LW were heavily polluted with Cr, Cu, Pb, and Zn. In contrast, the NF sediments were not considered as polluted with Cd, Cr, Cu, and Pb, but were only moderately polluted for Zn.

**Figure 2:**
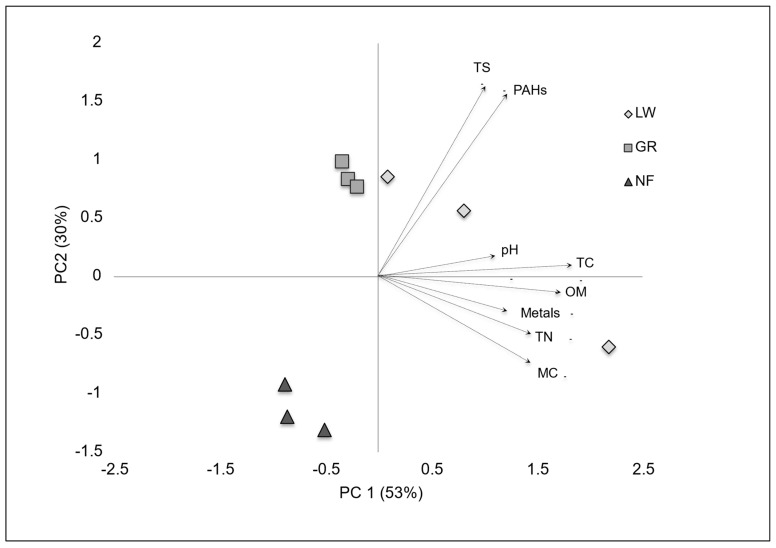
Ordination plot of principal component (PC 1 and PC 2) extracted from geochemical parameters pH, nitrogen (TN), carbon (TC), moisture content (MC), organic matter (OM), total PAHs, and total concentration of all metals. Shapes identify site data in sediments from Lowellville (diamonds), Girard (squares), and Newton Falls (triangles). Vector arrows on plot denote loadings of original geochemical variables.

One-way MANOVA of organic matter, pH, sulfate, nitrogen, total organic carbon, moisture content, total PAHs, and individual metal concentrations revealed that there were significant differences among the sites (Wilks’ Lambda = 0.000, *F* = 48.55, *p* = 0.020). MC, TC, total PAHs, metals, and sulfate exhibited statistically significant differences among the sites (*p* < 0.05). The scores and loadings of the principal components (Figure 2) for the three sites clearly discriminated by site, corroborating results from the MANOVA, which consistently identified site differences between environmental parameters. In addition, strong positive statistically significant Pearson correlations were found (Table 3) among MC and TC (r = 0.888, *p* < 0.01), OM (r = 0.793, *p* < 0.05), and metals (r = 0.877, *p* < 0.01). TN significantly correlated with MC (r = 0.929, *p* < 0.01), TC (r = 0.939, *p* < 0.01), OM (r = 0.927, *p* < 0.01), pH (r = 0.690, *p* < 0.05), and metals (r = 0.959, *p* < 0.01). TC strongly correlated with OM (r = 0.900, *p* < 0.01) and metals (r = 0.894, *p* < 0.01). OM strongly correlated with pH (r = 0.737, *p* < 0.05) and metals (r = 0.928, *p* < 0.01).

### 3.2. Bacterial Abundance

Enumeration of bacteria (by total counts) varied according to depth and site, ranging from 1.5 to 3.2 × 10^7^ in LW, 2.6 to 4.2 × 10^7^ in GR, and 2.6 × 10^7^ cells g^−1^ in sediment cores from NF. The abundance of bacteria did not correlate with sediment depth, and differences among cores and/or sites were not statistically significant (*p* > 0.05). However, SRB enumerated by MPNs, considered as a reliable method for bacterial estimation (Bhagobaty, 2014 [52]), revealed that the abundances of viable SRB varied individually per sediment core. In the sediment cores from LW, SRB ranged from 7.5 × 10^2^ to 1.1 × 10^4^, in GR from 2.4 × 10^3^ to 1.1 × 10^4^, and in NF from 2.1 × 10^2^ to 1.1 × 10^4^ cells g^−1^.

### 3.3. Microbial Community

**Figure 3:**
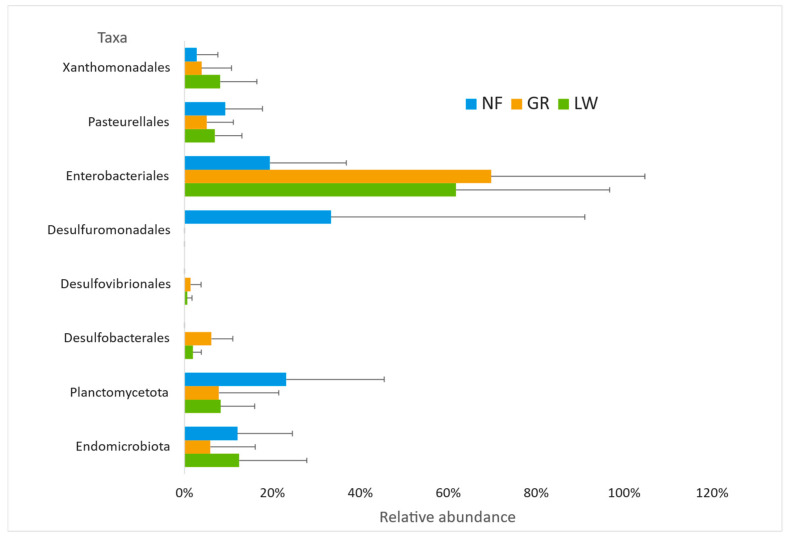
The relative abundance of bacteria (at the order level), recovered from next-generation sequencing (NGS) for the sediment samples at Lowellville (LW), Girard (GR), and Newton Falls (NF). Means and standard deviations are shown.

**Figure 4:**
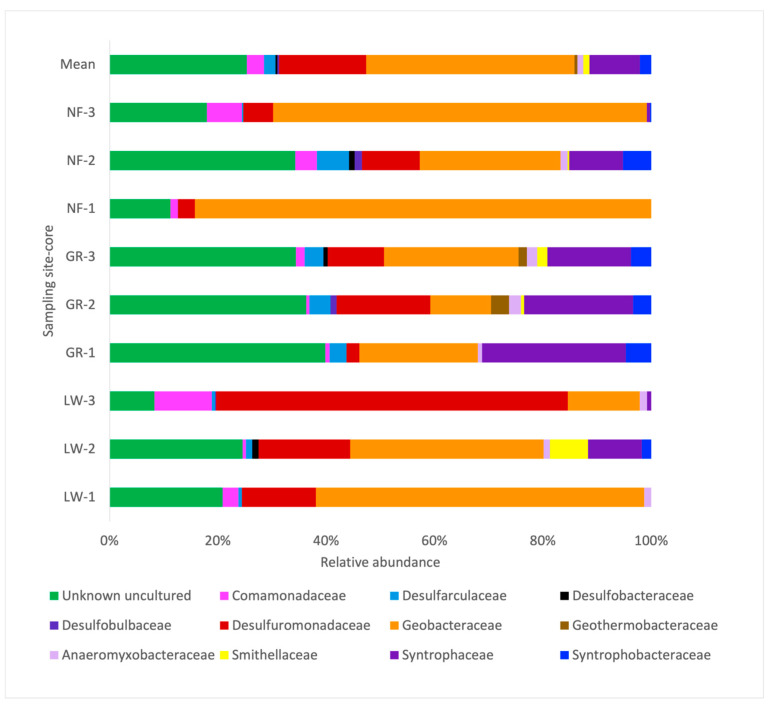
FeRB composition at the family level, recovered from next-generation sequencing (NGS) for the sediment samples at each core in Lowellville (LW 1–3), Girard (GR 1–3), and Newton Falls (NF 1–3).

The microbial composition and diversity were investigated by sequencing 16S rRNA amplicons, followed by NGS. Overall, the bacterial community exhibited great variability among the cores at each different sampling site (Figure 3). Alpha diversity was not statistically significant, yet some phylogenetic analyses identified that the bacterial community in LW was mainly composed of Thermoproteobacteria (79%), Elusimicrobiota (12%), and Planctomycetota (8%). At the order level, the community was represented mainly by Enterobacteriales, followed by Xanthomonadales and Pasteurellales (Figure 4). The GR sediment community was similar to the LW bacterial community, as it was also dominated by Thermoproteobacteria (87%), represented mainly by Enterobacteriales and Pasteurellales. Somewhat in contrast, the bacterial community in NF sediments, which showed the highest variability within cores, had in its composition fewer Thermoproteobacteria (32%) and more δ-Proteobacteria (33%). Moreover, several lineages involved in Fe transformation (i.e., *Geobacter* and *Stenotrophomonas*) and sulfur cycling (i.e., *Desulfovibrio*) species were also identified. Four dominant archaeal phylotypes represented in OTUs affiliated to the genera *Methanobacterium* and *Methanobrevibacter* were found in relatively deep sediments (~2.7 m), but only in sediments with a higher degree of contamination.

### 3.4. The Composition of the Iron-Reducing Community

**Figure 5:**
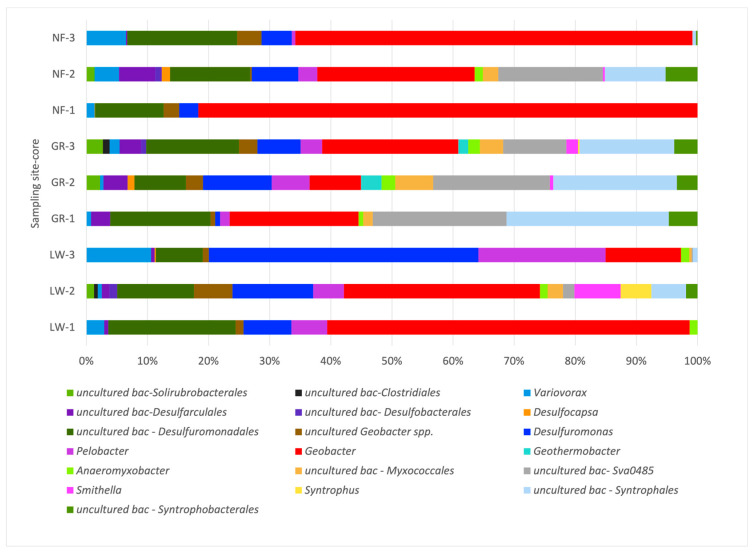
Iron-reducing bacteria (FeRB) composition at the OTU level, recovered from next-generation sequencing (NGS) for each sediment core at Lowellville (LW 1–3), Girard (GR 1–3), and Newton Falls (NF 1–3).

A closer look at the iron-reducing community in these metal-contaminated sediments was also investigated using NGS. The primers chosen yielded high-quality reads, performed well by generating few chimeras and high coverage (97% SILVA). Rarefaction curves of the FeRB indicated that sampling was enough to describe the diversity (corroborated by Good’s coverage estimator). FeRB members in the sediments collected from all three sites (LW, GR, and NF) belonged to the Thermodesulfobacteriota (~72%), followed by Pseudomonadota (21%), mainly represented by bacteria from the Desulforomonadales and Syntrophales, while a small percentage of sequences (<4%) corresponded to *Bacillota* and Actinomycetota. At the family level, bacteria belonging to Geobacteraceae comprised a high percentage of the community, ranging from 13 to 61% in LW, 11–26% in GR, and 26 to 84% in NF, followed by Desulfuromonaceae, representing 14 to 65% in LW, 2 to 17% in GR, and 3 to 11% in NF (Figure 4). In addition, Syntrophaceae representatives that carry the *hgcAB* gene involved in mercury methylation (Bravo et al. 2018 [53]) were present in sediments from each core at each sampling site. At the OTU level, spatial differences in composition were more evident (Figure 5). For example, the number of OTUs in LW ranged from 14 to 23, in GR from 16 to 28, and in NF from 10 to 24, yet the main dominant bacterium was *Geobacter*, which can utilize Fe (III), as well as MnO_4_ and AsO_3_, as electron acceptors in the degradation of organic matter (Wang and Pan, 2025 [25]) represented the highest relative abundance in all the sediment cores from LW (from 12 to 59%), followed by the sediments from GR (8 to 23%) and NF (26% to 88%). Similarly, *Desulfuromonas*, involved in dissimilatory iron reduction, represented the second highest relative abundance, ranging from 5% to 22% (Figure 4), followed by *Pelobacter* spp. (1% to 4%), also known as iron and sulfate reducers (Reyes et al. 2016 [54]). Phylotypes, including *Geothermobacter* and *Anaeromyxobacter*, a novel species with iron-reducing capabilities (Reyes et al. 2017 [55]), in addition to *Syntrophus,* were also found in smaller proportions. Other phylotypes belonging to Desulforomonadales and Myxococcales had an unknown affiliation (Figure 5).

**Figure 6:**
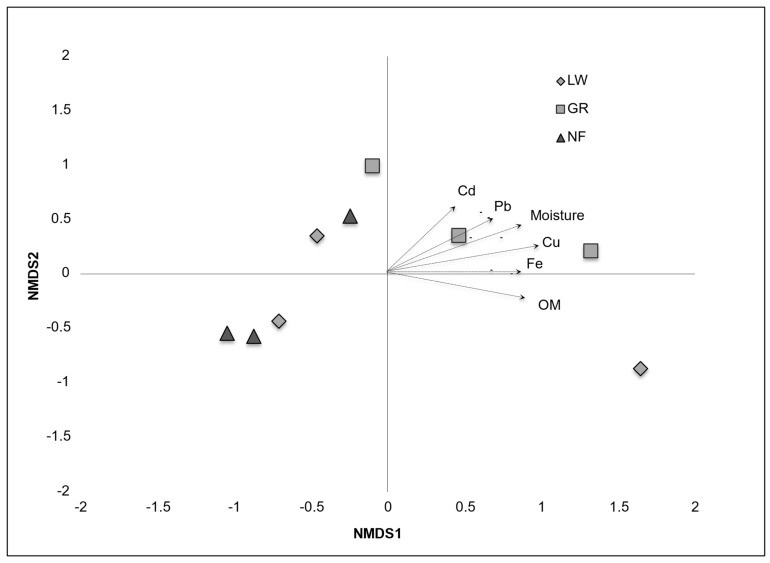
NMDS ordination showing similarity and distribution of FeRB, analyzed by Geobacteraceae gene sequences (NGSs) in riverbank sediments. The symbols represent the sites of Lowellville (diamonds), Girard (squares), and Newton Falls (triangles). The graph also shows ordination distances created using Spearman correlation values from the NMDS dimension scores of FeRB OTUs and geochemical variables: moisture content (MC), organic matter (OM), Pb, Cu, Cd, and Fe. Only significant correlations are shown.

Correlations between NMDS scores for FeRB gene sequences and geochemical parameters showed that sediments from GR, which had the least percentage of Geobacteraceae, appeared to be strongly influenced by some metals (Figure 6). The Shannon–Wiener index and Chao1 for FeRB were not statistically significantly different (*p* > 0.05) among cores and/or sampling locations (Table 4).

### 3.5. Molecular Diversity of dsrAB Genes

Limited information on the diversity of sulfate-reducing genes in communities with PAH–metal co-contamination is available. Dissimilatory sulfite reductase is a key enzyme in the process of anaerobic respiration of sulfite (Zhou et al. 2021 [56]); thus, both the alpha (*dsrA*) and beta (*dsrB*) subunits have been widely used for the detection of SRB (Zhu et al. 2022 [45]). In this study, partial *dsrAB* sequences were analyzed to identify sediment-associated SRB by clone library analysis. Selected screened sequences (pooled from nine cores) resulted in moderate to sufficient representation of the SRB community (Good’s coverage ranging from 23% to 86%), while the Shannon–Wiener *dsrAB* diversity index (H′) varied, ranging from 3.6 to 0.9, and was not statistically significant (*p* > 0.05). Most sequences were closely related to uncultured SRB without known affiliation (homology > 70%), followed by Thermodesulfovibrionales (19%) and *Nitrospirota* spp. (2%), two bacterial groups recently described in subsurface aquatic environments with low oxygen and sulfur-rich conditions (Mosley et al. 2024 [57]). In addition, a small percentage of members of Deltaproteobacteria (3%) and Desulfobacteraceae (1%) were also detected. Several sequences (6%) had a relatively low homology, perhaps indicating a divergent group of *dsrAB* genes (Zhu et al. 2022 [45]).

## 4. Discussion

The Mahoning has been exploited for over two centuries during a buoyant period of the steel industry in the northeastern US, before shutting down in the 1970s. Previous reports ranked the river as one of the most polluted in the world (Johnston et al. 2015 [4]), in which bacterial communities influenced by hydrocarbon contamination (Johnston and Leff 2015 [27]) harbored SRB (Pratt et al. 2012 [29]) and appeared to have potential for degradation of PAHs (Johnston et al. 2016 [28]; Acer et al. 2021 [58]). In addition, no previous published study has measured metal concentrations in these deep anoxic sediments of the riverbanks.

Conditions in riverine ecosystems are extremely variable in time and space. Riparian soil, groundwater, hyporheic zone, and water channel function in response to riverbank physico–chemical dynamics affecting microbial communities, which respond by modulating biogeochemical cycles. SRB and FeRB, groups involved in sulfur and iron transformations, are currently understudied in this type of ecosystem, and were assessed in this exploratory study in sites with different degrees of co-occurrence of PAHs and metal contamination.

### 4.1. Geochemical Footprints

Several studies have emphasized the importance of determining geochemical parameters as context for analyzing microbial communities (Gou et al. 2026 [7], Chen and Gu 2022 [59]). Geochemical heterogeneity was observed across cores and sampling sites, and, as found in other similar systems, higher PAH concentrations were present in deeper riverbank sediments, reflecting prior industrial activity and deposition processes over time along the riverbanks. Zhang et al. 2024 [6] reported similar observations from a total of nine sediment samples polluted with PAHs, metals, and PFAs, indicating that the highest PAH concentrations were found at deeper sediments. In contrast to other investigations, concentration of some metals, including Al, Mn, Zn and Pb, exceeded what has been reported for sites associated with past steel activities (Zhang et al. 2024 [6]; Zhang et al. 2022 [60]) and coking and steel production abandoned sites (Zhao et al. 2021b [61]), likely due to high total co-contamination of petroleum hydrocarbons associated with past intensive industrial activity, as previously described (reviewed in Johnston et al. 2015 [4]). Similarly, in this study, concentrations of sulfate (up to 8000 μM) were much higher compared to what is normally found in freshwater environments, ~300 μM (Pester et al. 2012 [62]), but still lower than marine sediments (Wunder et al. 2021 [8]). In this study, organic matter, PAHs, and metals appeared to influence community composition (strong correlation), yet might not be the causal factor, as concluded in other studies (Zhao et al. 2023 [63]). In fact, long-term exposure to PAHs and/or metals may lead to tolerance, favoring the abundance of some groups of bacteria better adapted to higher PAH–metal contamination (i.e., Thermodesulfobacteriota) compared to other groups (i.e., Planctomycota), which were more abundant in sediments with less contamination, an observation reported for similar systems (Gosai et al. 2022 [64]). In sedimentary systems with high sulfate concentrations (i.e., marine and salt marshes), methanogens can outcompete SRBs for substrate uptake (Sela-Adler et al. 2017 [65]). In this study, the few detected archaeal sequences were related to *Methanobacterium*, *Methanobrevibacter*, and several unknown members of the Methanobacteraceae; these groups were also found in other freshwater contaminated environments (Fan and Xing 2016 [66]).

### 4.2. Iron-Reducing Bacteria in High-Iron Environments

In anoxic environments, it is well-known that FeRB in the Geobacteraceae family oxidize organic matter and hydrogen while reducing oxidized metals and are also able to mineralize persistent aromatic hydrocarbons, particularly when iron oxides are abundant (Zhao et al. 2026 [1]). In Mahoning riverbank sediments, as anticipated due to the high levels of iron, members belonging to the Geobacteraceae and Desulfuromonadaceae were detected, representing 73% of the iron-reducing community. What might be unique to this system is that *Geobacter* spp. made up a large proportion of the Geobacteraceae (ranging from 17 to 57%). Interestingly, and concurrent with other studies (Vigderovich et al. 2019 [67]), *Desulfuromonas*, another Desulfuromonadaceae member involved in toluene degradation and iron oxide reduction (Kim et al. 2014 [68]), and *Pelobacter* spp., fermentative organisms with iron-reducing capabilities (Lovley et al. 1995 [69]), also represented a major proportion of the FeRB community in these sediments (Figure 5). Other studies have reported much lower percentages of *Geobacter* species. For instance, in petroleum-polluted iron-rich aquifers, *Geobacter* represented only 0.38% (Di et al. 2025 [15]), while in polluted eutrophic lake sediments, *Geobacter* accounted for 1.77% (Fan et al. 2018 [14]). In a detailed study, where uranium (VI) contaminated sediments were stimulated under iron reduction (with the addition of acetate), *Geobacter* species accounted for 35% of the microbial community at the end of the treatment, compared to only 5% in controls (mimicking natural *conditions*) where U (VI) and Fe (III) were not stimulated (Holmes et al. 2002 [37]). The presence of such a high amount of Fe (exceeding what has been reported in the literature on riparian sediments), mostly in the form of magnetite (Farnham et al. 2012 [30]), suggested that perhaps microbes in these sediments might use Fe as a terminal electron acceptor.

### 4.3. Sulfate-Reducing Bacteria and the Cryptic Cycle

SRB are essential for the global sulfur cycle, metal immobilization and removal (Ayangbenro et al. 2018 [70]), and can syntrophically grow with methanogens (Mills et al. 2016 [71]). SRB reduces the bioavailability of toxic heavy metals (e.g., cadmium, cobalt, and chromium) by transforming dissolved metal sulfates into insoluble metal sulfides (Zampieri et al. 2020 [11]). In uncontaminated saline and hypersaline lake sediments, SRB ranges from 10^2^ to 10^8^ cells/g (Foti et al. 2007 [72]). This current study placed the numbers of culturable SRB (10^4^ cells/g) within the same range as found in highly uranium-contaminated sediments (Sitte et al. 2010 [73]), and marine sediment contaminated with crude oil (Suárez-Suárez et al. 2011 [74]). These numbers are somewhat lower than what has been reported for surface sediments with much lower heavy metal concentrations (10^5^–10^8^ cells/g; Zhang et al. 2016 [75]). High metal contamination in Mahoning sediments, where Fe, Al, Zn, and Cu concentrations were 10 to 100 times higher than what has been reported in other polluted systems (Zampieri et al. 2020 [11]; Gou et al. 2026 [7]), might have selected for metal resistance (not measured). Further analyses of the dissimilatory sulfate reduction gene indicated that the majority of *dsrAB* sequences detected were related to unknown SRB reported in other contaminated environments (Zhu et al. 2022 [45]). Some of these sequences were related to Thermodesulfovibrionales, a novel group with potential for indirect bioremediation via formation of insoluble iron/sulfide minerals, which can adsorb or coprecipitate arsenic (Chen et al. 2024 [76]).

Recent studies (involving enrichment and isolation) have suggested that (at least in marine sediments) sulfate-reducing bacteria contributed to iron reduction (reviewed in Chen et al. 2025 [21] and references therein). Many dissimilatory sulfate-reducing bacteria have since been shown to utilize sulfate and/or other alternative electron acceptors, including metal oxides, for anaerobic respiration (Reyes et al. 2017 [55]). The CSC, where both SRB and FeRB interact metabolically, can be difficult to detect because of direct and rapid coupling to the reduction of metal oxides, mainly iron oxides (Nghiem et al. 2023 [77]). Although direct quantification of microbial sulfate reduction provides the best proof of active sulfur cycling (Holmkvist et al., 2011 [78]), it can be inferred that culturable SRB in Mahoning riverbanks might be participating in cryptic sulfur cycling. Indirect supportive findings include enumeration of SRB, the presence of the *dsrAB* gene sequences, the higher abundance of *Geobacter* spp., and extreme levels of sulfate and iron.

### 4.4. Limitations of the Study

The majority of environmental research, performed under strict laboratory conditions using microcosms and/or bench assays, overcomes several constraints (e.g., lack of representativeness of the natural process, site access, heterogeneity, and maintaining an oxygen-free environment). However, working in situ through these same limitations represents a complementary approach to inquire how microbes interact with their surroundings. In this study, the nature of sediment with high amounts of organic matter and total petroleum (not measured in the study) did not always yield good-quality DNA, compromising further genomic analyses (i.e., archaeal library). The SRB community clone analysis was moderately sampled, though this was somewhat compensated for, since Sanger sequencing has inherently lower sequence error rates than NGS. Rates of sulfate and/or iron reduction were not monitored. Thus, this study should be taken as an exploratory investigation. More extensive research in this system is warranted for a detailed and precise determination of sulfate and iron cycling, and how co-contamination affects the structure and function of these two microbial communities.

## 5. Conclusions

Riparian zones that converge with contaminated riverbank sediments represent a unique microbial habitat important to nutrient cycling and pollutant degradation. Analyzing the mechanisms by which microbial communities survive under stress conditions was beyond the scope of this work. However, using moderate- to high-resolution molecular techniques, overall, SRB and FeRB microbial communities in PAH/metal co-contaminated Mahoning riverbank sediments suggested both communities might be interacting actively as potential participants in a CSC, given the high sulfate and iron concentrations. Thus, a pool of terminal electron acceptors (i.e., sulfate and iron oxides) with a resupply mechanism (CSC), the presence of taxa capable of anaerobic respiration, and high levels of PAHs (which can act as carbon sources), combined with previous work in this system (showing high microbial biomass and activity in the most heavily contaminated sediments), indicate that essential components are all present for a potential bioremediation strategy. Future experiments on how these two important groups of bacteria control the biogeochemistry of an altered riverine system are warranted.

## Figures and Tables

**Table 1:** Geochemical parameters of riverbank sediments, moisture content (MC), sulfate, total nitrogen (TN), organic matter (OM), total carbon (TC), total PAHs (∑PAHs), depth, and pH.

Site	Core	Latitude/Longitude	MC%	SO_4_^−2^ (μM)	TN%	OM%	TC%	∑PAHs	Depth (m)	pH
Lowellville	LW1	41.038450, −80.541306	38.2	8754.4	0.2	9.1	8.9	416,290.2	2.7	6.9
	LW2	41.038253, −80.540208	42.9	3463.8	0.2	8.5	10.2	218,037.4	2.4	7.2
	LW3	41.037950, −80.539464	36.0	5058.8	0.2	11.5	6.9	122,275.0	2.1	7.3
Girard	GR1	41.155017, −80.706528	50.2	822.9	0.2	7.4	7.7	95,195.9	1.2	6.8
	GR2	41.154714, −80.706450	52.7	336.1	0.2	9.5	8.7	79,484.9	1.5	7.0
	GR3	41.154553, −80.706389	47.3	771.5	0.2	9.3	8.3	107,072.4	2.1	7.2
Newton Falls	NF1	41.135164, −80.967211	20.4	1291.4	0.1	1.8	1.0	102,451.8	2.1	6.9
	NF2	41.135486, −80.967144	25.3	2298.7	0.1	2.5	1.8	51,347.3	1.2	6.0
	NF3	41.135644, −80.967067	22.2	1326.2	0.0	1.6	0.5	56,622.3	1.5	6.6

**Table 2:** Metal concentrations expressed in μg g^−1^ dry weight found in riverbanks from Lowellville (LW), Girard (GR), and Newton Falls (NF) along the Mahoning River in each sediment core.

Site			Metal Concentrations																			
	Core		Be	Al	Cr	V	Mn	Fe	Co	Ni	Cu	Zn	Ga	As	Se	Sr	Cd	Ag	Ba	Pb	Tl	U
Lowellville	LW1	Mean	1.5	13,047.8	175.6	63.8	7703.3	320,968.1	36.5	84.7	423.5	1157.0	5.7	32.8	3.4	53.7	3.4	5.2	118.8	349.5	3.5	1.5
		SD	0.9	594.1	23.3	6.2	102.6	5863.3	2.3	11.5	58.5	193.8	0.3	4.1	0.2	3.9	0.3	1.0	15.1	61.2	0.8	0.5
	LW2	Mean	0.8	15,007.6	231.4	54.8	6903.7	236,704.6	29.0	87.1	403.1	1782.1	5.1	22.0	4.3	54.1	3.1	7.1	135.7	441.3	4.2	1.1
		SD	0.3	2453.0	28.1	4.0	83.9	16,046.5	1.8	9.9	69.9	578.1	0.5	3.1	0.9	3.1	0.6	1.3	12.4	133.9	0.3	0.2
	LW3	Mean	0.2	6886.9	215.5	43.7	8623.5	344,115.3	33.8	98.9	437.2	949.5	3.7	14.1	0.5	48.9	1.4	2.5	78.8	226.2	1.9	0.6
		SD	0.2	1175.4	26.3	4.8	1458.0	50,206.3	3.5	9.8	102.6	213.9	0.5	1.7	0.5	2.7	0.7	1.1	24.4	49.6	0.7	0.1
Girard	GR1	Mean	0.8	11,664.7	728.7	80.4	6575.1	380,035.7	56.9	281.9	1641.0	1729.4	8.8	72.1	3.5	41.6	6.2	15.1	135.2	431.9	4.1	1.9
		SD	0.2	1970.3	220.4	8.4	462.7	16,811.3	2.6	44.5	78.0	103.2	0.3	10.4	1.8	4.5	0.7	1.0	15.0	16.0	0.4	0.3
	GR2	Mean	0.7	14,562.3	712.3	81.9	5975.8	289,943.1	40.3	347.7	1225.0	2511.9	7.0	48.1	2.2	57.4	7.4	20.5	142.7	500.8	3.5	1.7
		SD	0.3	1467.5	12.8	3.1	443.4	26,126.4	1.6	9.0	31.0	98.1	0.1	1.8	3.4	4.2	0.6	0.4	4.3	14.4	0.2	0.2
	GR3	Mean	1.0	14,211.0	631.5	99.2	6551.9	315,851.4	51.5	284.1	1540.0	2246.1	8.3	59.1	0.7	51.9	8.7	15.9	152.8	420.5	4.6	4.7
		SD	0.3	2560.7	88.5	13.7	1363.3	8608.9	2.6	35.4	190.2	264.4	0.7	7.0	1.7	10.1	2.2	0.8	30.8	40.0	1.2	0.9
Newton Falls	NF1	Mean	0.3	7548.9	19.7	37.5	1725.8	16,820.5	21.4	14.2	23.8	164.5	5.0	11.8	0.0	10.2	0.2	0.4	63.9	10.0	0.5	0.6
		SD	0.2	807.8	3.1	3.9	531.2	2169.7	2.0	1.3	2.4	36.1	0.5	2.1	0.7	0.9	0.2	0.2	9.3	0.8	0.2	0.2
	NF2	Mean	0.7	6475.9	19.6	30.2	427.1	11,831.6	13.4	10.5	21.8	192.3	3.9	6.1	0.6	5.9	0.6	0.4	39.8	8.8	0.8	0.5
		SD	0.7	1211.4	1.1	3.8	41.1	1052.5	1.4	1.1	3.9	131.8	0.2	0.6	1.4	0.4	0.2	0.5	2.9	1.6	1.0	0.1
	NF3	Mean	0.4	7163.6	21.2	33.0	417.5	11,356.4	15.3	11.3	18.7	82.7	4.9	7.4	0.9	6.3	0.1	0.2	46.8	7.7	0.3	0.4
		SD	0.5	1886.8	3.7	8.3	60.7	1916.2	4.6	3.1	5.6	19.1	1.6	0.8	2.7	1.1	0.2	0.1	12.1	1.8	0.0	0.1

**Table 3:** Pearson correlations found between geochemical parameters, moisture content (MC), total nitrogen (TN), total carbon (TC), sulfate, pH, and depth measured in riverbank sediments from the three sampling sites (Lowellville, Girard, and Newton Falls). The significant correlation coefficients are underlined (double at the 0.01 level, and single at the 0.05 level).

	MC	TN	TC	Sulfate	pH	Depth
MC						
TN	0.929					
TC	0.888	0.939				
Sulfate	0.077	0.149	0.408			
pH	0.497	0.690	0.657	0.097		
Depth	0.022	0.266	0.425	0.663	0.652	
∑PAH	0.163	0.293	0.520	0.966	0.338	0.797
∑METAL	0.877	0.959	0.894	0.258	0.656	0.275

**Table 4:** Shannon–Wiener index [H′], Chao1, and Good’s estimator for FeRB in riverbank sediments calculated based on rarefied NGS sequences.

Site	Core	H′	Chao1	Good’s
Lowellville	LW1	4.29	41.9	87%
	LW2	4.76	58.1	80%
	LW3	3.62	33.5	90%
Girard	GR1	4.65	48.2	84%
	GR2	4.82	60.6	82%
	GR3	5.17	76.1	74%
Newton Falls	NF1	3.68	29.1	92%
	NF2	4.92	68.0	78%
	NF3	4.47	42.5	88%

## Data Availability

The original contributions presented in this study are included in the article. Further inquiries can be directed to the corresponding author.
